# TDP-43 in nuclear condensates: where, how, and why

**DOI:** 10.1042/BST20231447

**Published:** 2024-07-03

**Authors:** Ruaridh Lang, Rachel E. Hodgson, Tatyana A. Shelkovnikova

**Affiliations:** Sheffield Institute for Translational Neuroscience (SITraN) and Neuroscience Institute, University of Sheffield, Sheffield, U.K.

**Keywords:** ALS, condensate, neurodegeneration, nuclear body, paraspeckle, stress response, TDP-43

## Abstract

TDP-43 is an abundant and ubiquitously expressed nuclear protein that becomes dysfunctional in a spectrum of neurodegenerative diseases. TDP-43's ability to phase separate and form/enter biomolecular condensates of varying size and composition is critical for its functionality. Despite the high density of phase-separated assemblies in the nucleus and the nuclear abundance of TDP-43, our understanding of the condensate-TDP-43 relationship in this cellular compartment is only emerging. Recent studies have also suggested that misregulation of nuclear TDP-43 condensation is an early event in the neurodegenerative disease amyotrophic lateral sclerosis. This review aims to draw attention to the nuclear facet of functional and aberrant TDP-43 condensation. We will summarise the current knowledge on how TDP-43 containing nuclear condensates form and function and how their homeostasis is affected in disease.

## Introduction: TDP-43 and its compartment-specific condensation

TAR DNA-binding protein 43 (TDP-43) is an abundant, ubiquitously expressed RNA/DNA-binding protein (RBP) — member of the hnRNP family involved in RNA processing/metabolism and playing a crucial role in pre-mRNA splicing [1]. TDP-43 regulates a multitude of RNA targets, primarily RNAs with long introns enriched in the central nervous system (CNS) [2]. By binding to UG-rich motifs over-represented in introns, TDP-43 regulates alternative splicing [3], including cryptic exon repression [4] and alternative polyadenylation [5]. Like many other RBPs, TDP-43 controls its own expression at the RNA level [6]. In addition to splicing, the protein is involved in microRNA biogenesis [7] and RNA transport [8], as well as restricts retrotransposon activity [9].

TDP-43 is a component of multiple biomolecular condensates, or membraneless organelles (MLOs) [10]. MLO biogenesis relies on biological phase separation, including its liquid-liquid variant (LLPS) [11–13]. Similar to other RBPs, TDP-43 is capable of phase transitions, driven by specific structural features such as the low-complexity domain (LCD), enabling its partitioning into MLOs [14]. High RNA content in the nucleus creates an ideal environment for the biogenesis of MLOs — nuclear bodies, of which most are RNA-rich assemblies [15]. Indeed, RNA is a nucleating agent for many condensates, and the RNA-binding specificity of TDP-43 shapes/fine-tunes the properties of the condensates it enters [3,16]. Within the condensate, TDP-43 can engage in different intermolecular interactions conferring specific material properties, in order to meet the MLO's functional requirements [16,17].

TDP-43 is mostly nuclear in the steady-state [18], with its active nucleocytoplasmic transport mediated karyopherins, karyopherin-α (KPNA) and -β1 (KPNB1) [19]. Within the nucleus, a punctate, non-homogeneous pattern of TDP-43 distribution was first noted two decades ago [20]. Experimental evidence supports TDP-43 association with and physiological roles in the constitutive nuclear bodies paraspeckles [21], Cajal bodies (CBs)/Gems [22,23] and promyelocytic leukaemia protein (PML) bodies [24]. Furthermore, a global TDP-43 redistribution between nuclear bodies, alongside the assembly of *de novo* TDP-43 rich condensates, occurs in stressed cells [25–27]. TDP-43 can shuttle out of the nucleus, and despite its low cytoplasmic levels in healthy cells, varying levels of TDP-43 are detectable in stress granules, P-bodies, axonal granules and myo-granules. Within these assembles, TDP-43 contributes to RNA triage, protection and transport, as discussed extensively elsewhere [28–30]. TDP-43's cytoplasmic retention/accumulation has been linked to several common neurodegenerative diseases, having been observed in postmortem specimens and replicated in numerous disease models [31,32]. Although a host of studies have addressed the (phase-separating) behaviour of TDP-43 in the cytoplasm, its higher-order assembly in the nucleus is only now coming into the limelight. There is also a growing appreciation that the earliest alterations to TDP-43 metabolism in neurodegenerative disease occur in the latter cell compartment.

This review will focus on the nuclear facet of TDP-43 condensation, summarising and discussing the mechanisms and consequences of TDP-43 partitioning into nuclear MLOs in health and disease.

## Molecular mechanisms of TDP-43 condensation in the nucleus

Biological phase separation is driven by a summation of multiple weak interactions e.g. electrostatic, hydrogen bonds and cation-π interactions, and heavily relies on the multivalency of the molecules involved in the process [12,33]. Structurally, RNA, being a long, flexible charged polymer, is ideally suited to promote RBP condensation, and LLPS in particular [34,35]. RBPs have a modular structure also highly amenable to initiating and supporting phase separation, including via multivalent RNA binding [36,37]. Many RBPs contain an RNA recognition motif(s) (RRM) and a disordered, LCD rich in aromatic and polar amino acids and prone to self-association [38].

### TDP-43 structure

**Figure 1.**
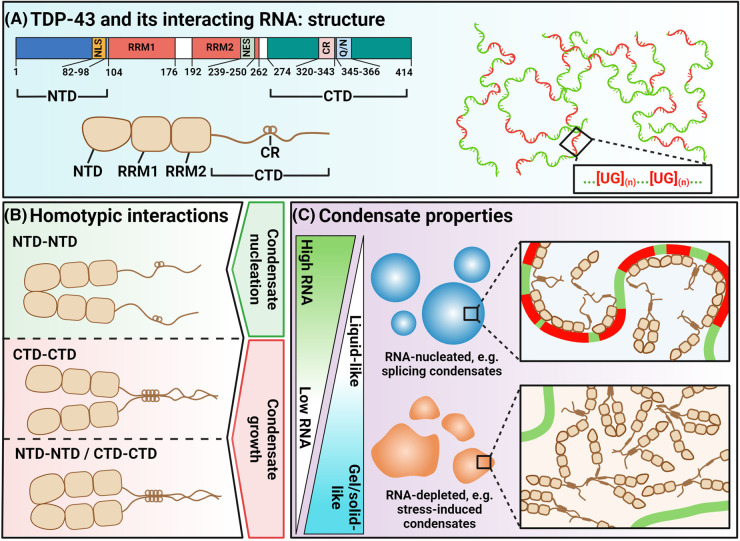
Molecular determinants and mechanisms of TDP-43 condensation. (**A**) TDP-43 domain structure and interacting RNA species. (**B**) Homotypic TDP-43 interactions and their role in nuclear condensate assembly. (**C**) Properties of TDP-43 containing nuclear condensates depending on the RNA content and types of homotypic interactions. *References for all figures are provided in the main text or* Table 1.

TDP-43 comprises an N-terminal domain (NTD), two RRMs and a C-terminal domain (CTD). The CTD of TDP-43 is represented by a glycine-rich LCD, ∼80% of which is intrinsically disordered [39–41] (Figure 1A). A unique feature of TDP-43's CTD is the presence of an evolutionarily conserved α-helix, known as ‘conserved region’ (CR, aa. 320–343), critical for protein-protein interactions and LLPS [42,43]. In addition, four ‘LARKS’ (low-complexity aromatic-rich kinked segments), that engage in amyloid-like labile interactions and contribute to physiological phase separation, have been identified in the CTD [41]. RNA-binding domains of TDP-43 are represented by two tandem RRM motifs [1,44]. Each RRM can bind 5xUG repeats [45], with longer UG stretches promoting binding cooperativity and phase separation [46,47]. On its N-terminus, TDP-43 contains a well-folded domain essential for its multimerisation [48–50]. Finally, the nuclear localisation signal of TDP-43 adjacent to RRM1 also modulates phase separation [51].

**Table 1.** TDP-43 containing nuclear condensates: properties and function

Nuclear condensate	Properties; homotypic TDP-43 interactions	RNA requirement/RNA type	Main cellular function	TDP-43 role in condensate	References
Constitutive					
‘Splicing condensate’	Highly dynamic (1,6-HD sensitive, dynamic by FRAP); CTD/CR-dependent	RNA-nucleated: length- and sequence-dependent, >100 nt regions, sparse UG motifs; multiple target RNAs	Pre-mRNA splicing	Condensate nucleation; TDP-43 is main component	[16]
Paraspeckle	Highly dynamic (1,6-HD sensitive, dynamic by FRAP); NTD-dependent	RNA-nucleated: UG-rich regions of NEAT1_2; other RNAs – AU-rich, pri-miRNAs	Gene expression regulation, including under stress; late response to stress	Condensate suppression: maintaining low basal assembly; TDP-43 is a negative regulator	[27,59,75,78,79,86,87]
Cajal body/Gem	Highly dynamic 1,6-HD sensitive, dynamic by FRAP); CR/CTD- and NTD-dependent	Rich in RNA: snRNAs, scaRNAs, etc.	Spliceosome biogenesis (U snRNP assembly)	Condensate integrity: maintaining snRNA/snRNP levels	[22,23,91–93,133,134]
PML body	Relatively dynamic (FRAP); TDP-43 can transition into solid-like state with longer stress	RNA-depleted but can sequester certain RNAs	Stress response; RBP protection from misfolding and degradation under stress	N/A (regulated by PML body)	[24,135]
Stress-induced					
Stress-induced nuclear *de novo* TDP-43 condensate (TC)	Solid-like (1,6-HD resistant, low dynamics by FRAP, stains for amyloid); NTD- and CTD-dependent	RNA-depleted but can sequester certain RNAs; negatively regulated by (UG-rich) RNA	TDP-43 sequestration/ inactivation; regulation of TDP-43 mediated splicing under stress	Condensate nucleation; TDP-43 is main component	[26,52,53]
Nuclear stress body (nSB)	Solid-like? CTD-dependent	RNA-nucleated: satellite III lncRNA; depleted of TDP-43 target RNAs	RNA processing under stress	n.d.	[25]
Interleukin splicing activating compartment (InSAC)	Solid-like? TDP-43 is ubiquitinated	Contains interleukin-encoding pre-mRNAs	Immune-related RNA processing	RNA sequestration and processing	[103]
Nucleolar cap	Both liquid- and solid-like subdomains	RNA-depleted but contains pre-rRNA transcripts	TDP-43/RBP protection from degradation? Nucleolus-related functions	n.d.	[79,104]

### Contribution of N-terminal and C-terminal domains

Most insights into the modes of TDP-43 condensation in the nucleus have come from *in vitro* studies with purified recombinant proteins. Several key amino acid residues in the extreme TDP-43 NTD were found to enable its dimerisation, and six point mutations abolish its NTD-driven self-assembly and CB partitioning [23,50]. However, even a subtle structural change in this domain (two point mutations), is sufficient to compromise TDP-43 association with nuclear MLOs, for example, partitioning into paraspeckles or biogenesis of stress-induced TDP-43 rich condensates [27,52]. Homotypic interactions via the CTD are equally important for TDP-43's higher-order assembly in the nucleus, e.g. partitioning into ‘splicing condensates’, CBs/Gems and stress-induced assemblies [16,22,25,53]. CR specifically was found to be important for TDP-43 coalescence into ‘splicing condensates’ [16] and Gems [22]. Interactions between other CTD segments, for example via LARKS, also contribute to association with nuclear bodies [41]. Indeed, CR deletion does not completely abolish Gem recruitment [22]. It is plausible that the NTD of TDP-43 plays a pivotal role in initiating condensation, whereas the CTDs brought into proximity by NTD-NTD interactions propagate phase separation and maintain condensate growth [14,54] (Figure 1B; Table 1). For instance, the intact NTD is required for the nucleation of TDP-43 stress-induced condensates, however they are also detected by a conformation-specific antibody that recognises only non-NTD-oligomerised TDP-43 species [26,52]. A switch from NTD-NTD to CTD-CTD interactions, alongside remodelling of CTD-CTD interactions, may enable an amyloid-like, β-sheet arrangement and relative stability of physiological TDP-43 condensates [41,52,53,55]. Further cellular studies are warranted to establish how the stoichiometries of different homotypic TDP-43 interactions are maintained and elucidate their relative contribution and interplay in different nuclear condensates, both in the steady state and under stress (Figure 1B).

### RNA-nucleated condensation

**Figure 2.**
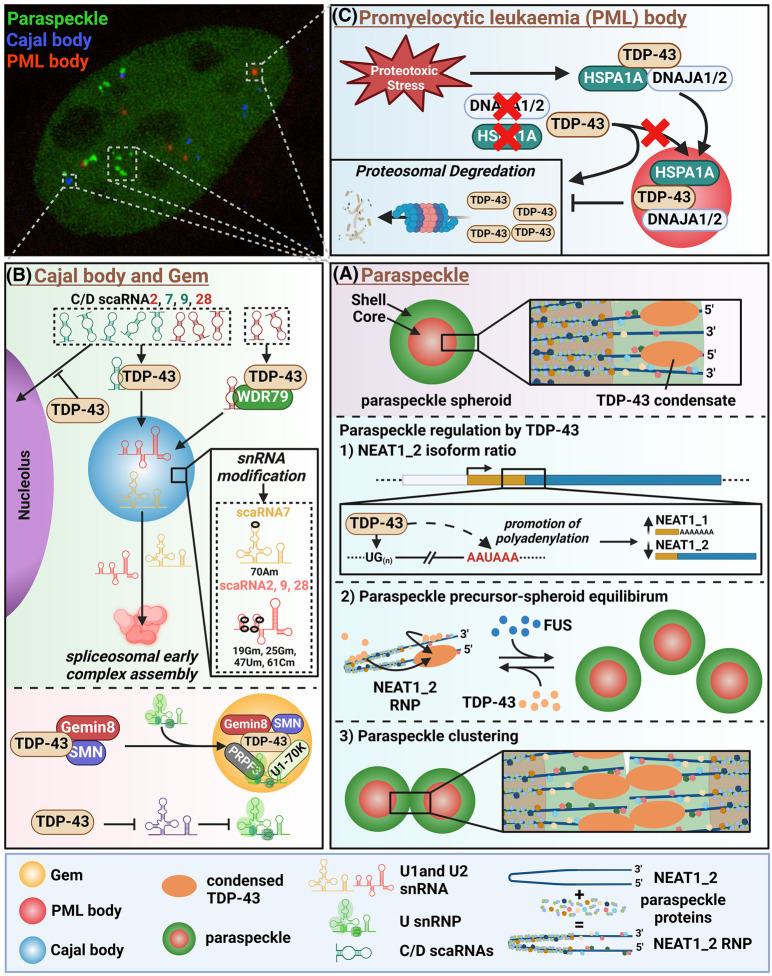
Constitutive TDP-43 containing nuclear condensates. (**A**) Regulation of paraspeckles by TDP-43. (**B**) TDP-43 roles in Cajal body/Gem maintenance and spliceosome. (**C**) Regulation of TDP-43 by PML bodies during stress. In the microphotograph of the nucleus, paraspeckles are visualised using NONO-GFP expression; Cajal bodies and PML bodies were visualised using anti-coilin and anti-PML staining, respectively.

RNA is the main structural component of most nuclear condensates [56] and one of the well-established TDP-43 LLPS drivers [57]. *In vitro* and cellular studies have demonstrated the dynamic nature of TDP-43/RNA complexes, where RNA acts as a molecular chaperone supporting their fluidity and liquid-like properties [46,47,58] (Figure 1C). RNA binding is important for TDP-43 partitioning into CBs/Gems, which is attenuated by TDP-43 RRM1 deletion or point mutations [22,23]. RNA-seeded TDP-43 self-assembly in the nucleus yields ‘splicing condensates’ — submicroscopic LLPS droplets on UG-rich intronic sequences [16]. The UG-rich motif composition dictates the condensation patterns of TDP-43 on RNA, where ‘splicing condensates’ form primarily on UG-repeat sequences dispersed within relatively long, >100 nt, regions [16]. Another example is the RNA-nucleated TDP-43 self-assembly on UG repeats in NEAT1_2 lncRNA that enables its partitioning into paraspeckles [21,27,59] (Figures 1C and 2A). NEAT1_2 UG-repeat driven condensation of TDP-43 also achieves its specific intra-MLO localisation — enrichment in the paraspeckle ‘shell’ [27]. Most intriguingly, the shell localisation of TDP-43 does not depend on UG-repeat positioning in the NEAT1_2 molecule, instead, TDP-43 is displaced from the repeats after nucleation and relocated from core to shell; another RBP, FUS, may play a role in this displacement [27] (Figure 2A). These findings implicate other factors, such as competition between different RBPs, in the modulation of RNA-seeded TDP-43 LLPS in the nucleus.

### RNA-independent condensation

RNA-depleted nuclear condensates are on the other end of the spectrum (Figure 1C; Table 1). Loss of interactions with RNA is often considered pathological for TDP-43, since in its RNA-unbound form, TDP-43 is prone to fibrillisation, sometimes irreversibly [47,58,60]. Nuclear stress bodies (nSBs) [25] and stress-induced *de novo* TDP-43 condensates [52,53] are the two examples of physiological RNA-depleted MLOs rich in TDP-43 whose assembly is accompanied by TDP-43 dissociation from RNA. Strikingly, although the TDP-43 CTD in isolation readily undergoes LLPS [14], the full-length recombinant protein is prone to forming solid-like assemblies under RNA-free conditions *in vitro,* unless solubilisation tags are utilised [47,61]. Thus, in the absence of RNA chaperoning, full-length TDP-43 is prone to homotypic interactions yielding structures with reduced liquid properties.

‘Splicing condensates’ and stress-induced *de novo* TDP-43 condensates, being RNA-seeded liquid-like and RNA-depleted solid-like assemblies, respectively [16,59], represent the two extremes in the continuum of RNA enrichment and fluidity (Figure 1C). However other nuclear bodies may not be as easily classifiable into RNA-enriched and RNA-depleted, and into LLPS-based and non-LLPS/solid-like. Instead, within different nuclear condensates, TDP-43 may form more and less liquid subdomains composed of RNA-bound and RNA-unbound species, respectively, which defines the properties of the condensate as a whole. The aliphatic alcohol 1,6-hexanediol has been instrumental in probing the nature of biomolecular condensates [59,62,63] however its use has limitations in live cells [64]. A combination of approaches, including FRAP, aliphatic alcohol exposure and single-molecule imaging approaches [65], will be required to delineate the molecular states of TDP-43 within nuclear condensates (Figure 1C).

### Post-translational modifications

Post-translational modifications (PTMs) represent another layer in the tight control of TDP-43 condensation behaviour and nuclear condensate association [66]. Modification of a single amino acid residue can have a dramatic effect on TDP-43 phase separating properties, underscoring the importance of this regulatory mechanism in cells. For example, serine phosphorylation both in the NTD and CTD modulates TDP-43 homotypic interactions and LLPS [67]. Serine-48 is a critical residue in the TDP-43 NTD whose phosphorylation impacts the protein's self-assembly and phase separation [68]. Although the effects of this PTM on nuclear MLOs are yet to be pinpointed, a negative effect on splicing suggests a disruption of condensation. CTD (hyper-)phosphorylation was found to reduce its stress-induced condensation and render TDP-43 condensates more liquid-like, without changes in RNA binding [61]. On the other hand, lysine acetylation in RRMs (K136/145/192) is sufficient to disrupt its RNA binding and induce nuclear TDP-43 aggregation [60,69,70].

Thus, TDP-43 condensation within different nuclear MLOs proceeds via different routes, where homotypic TDP-43 interactions are modulated by RNA, PTMs and other RBPs, translating into a range of physical states and functionalities.

## TDP-43 in constitutive nuclear condensates

### Splicing condensates

Earlier findings on the contribution of TDP-43 self-association to its splicing competency were contradictory. TDP-43 autoregulation or target gene splicing were found to be compromised by NTD or CTD mutations impacting phase separation in some reports [43,50,71,72] but not in others [73,74]. However, these results can be reconciled within the recently proposed ‘binding-region condensate’ model, where only certain splicing targets are affected by disruption of (CTD-driven) TDP-43 condensation [16]. The RBPchimera-CLIP approach revealed that only the targets containing sparsely positioned YA[UG]n motifs within longer regions (>100 nt) rely on intact TDP-43 condensation for their splicing; consistently, TDP-43 binding to such motifs is sensitive to 1,6-hexanediol. In contrast, splicing of targets with more dense arrangement of TDP-43 binding motifs within a shorter region does not require TDP-43 condensation. Such sub-nanometre TDP-43 condensates may play roles in other nuclear RNA metabolism processes, beyond splicing, which is yet to be investigated.

### Paraspeckles

Paraspeckles are phase-separated nuclear bodies broadly involved in gene expression regulation via sequestration of RNAs and proteins [75,76]. This MLO is assembled around the ‘architectural’ nuclear-retained lncRNA NEAT1_2 — the most reliable paraspeckle marker — and possesses an internal core-shell substructure [77]. Multiple RBPs, firstly, NONO and SFPQ, are recruited onto this RNA scaffold, to form NEAT1_2 RNPs – paraspeckle precursors, which are then joined together into a ∼350 nm spheroid by FUS protein [78,79]. FUS plays a pivotal role in the spheroid assembly; paraspeckles spheroids as such represent FUS condensates. Paraspeckles are absent from the majority of healthy mammalian tissues [80], and their assembly is triggered by normal developmental programmes [21] and stress [81,82], but also by tumorigenesis [83] and neurodegeneration [84]. Strictly speaking, paraspeckles are stress-inducible condensates, however, they are present in nearly all proliferating mammalian cell lines in culture, except embryonic stem cells [85], suggesting that culturing conditions represent a type of stress state requiring their constitutive maintenance [86].

**Figure 3.**
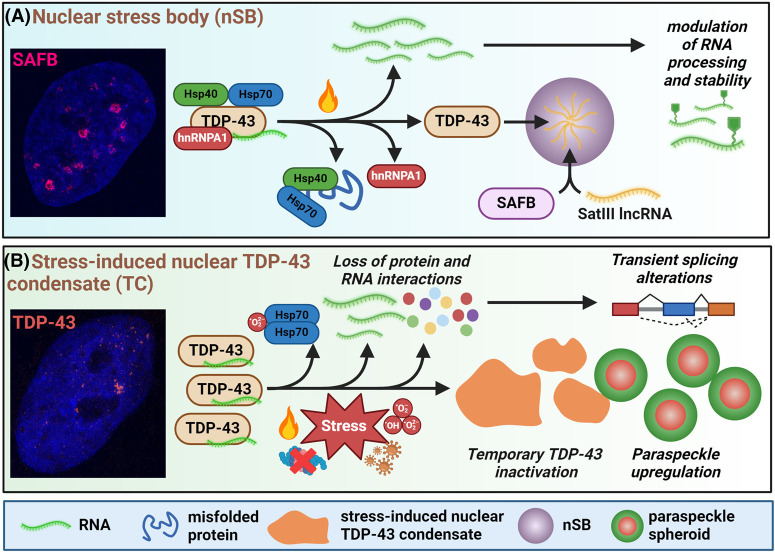
Stress-inducible TDP-43 containing nuclear condensates. (**A**) Regulation of TDP-43 interactions and availability by nuclear stress bodies (nSBs). (**B**) Stress-induced TDP-43 condensation in the nucleus leading to TC assembly, splicing alterations and paraspeckle up-regulation. In B, stresses are heat shock, proteasome inhibition, oxidative stress, and viral infection.

TDP-43 has been found to negatively regulate paraspeckles by promoting NEAT1 polyadenylation, thereby preventing the production of NEAT1_2 [21]. Unlike other RBPs, TDP-43 localises to the paraspeckle outer shell [77] in the form of micro-condensates [27] (Figure 2A). Its recruitment into NEAT1_2 RNPs is mediated by UG-rich tracts (four repeats ≥8 units in humans) [21] (Figure 2A). However the functional significance of TDP-43 binding to the paraspeckle precursors and its spheroid shell localisation remained enigmatic. Our most recent studies have allowed to link these features to the paraspeckle regulation, downstream of NEAT1 isoform processing. Using super-resolution imaging, NEAT1 gene editing and cell lines with different precursor-spheroid stoichiometry, TDP-43 has been found to act in preventing FUS-mediated condensation of paraspeckle precursors into the mature spheroid. This was also recapitulated with *in vitro* condensates of recombinant FUS protein. Furthermore, TDP-43 presence in the spheroid shell promoted the clustering of spheroids, which may restrict their intra-nuclear mobility and decrease functionality [27] (Figure 2A). Given the constitutive activity of the *NEAT1* promoter [80], such a three-tier control mechanism is seemingly vital for ensuring efficient paraspeckle suppression in the steady-state. On the other hand, during stress, TDP-43 sequestration into *de novo* condensates would alleviate paraspeckle inhibition and enable a burst of their assembly (Figure 3). Of note, TDP-43 may also have other functions within paraspeckles, e.g. in miRNA processing [87] or RNA/protein retention [75].

### Cajal bodies/Gems

CBs and gemini of coiled bodies (Gems) are small, <1 µm nuclear MLOs serving as sites for assembly and modification of small nuclear ribonucleoproteins (snRNPs) critical for pre-mRNA splicing [88]. Although their numbers vary, they are found in nearly all cell types, representing a prototypical constitutive nuclear body. CBs and Gems are merged into one structure in some cell types but are separate in others — a phenotype regulated by methylation of tudor-domain proteins [89]. Coilin (p80) is a reliable CB marker, whereas anti-SMN staining is used to visualise Gems. Unlike paraspeckles, CBs/Gems are typically dispersed during stress [90].

TDP-43 co-localisation with SMN and Gemin 8-positive structures in the nucleus was observed, with varying co-localisation with coilin-positive CBs, depending on the cell type [22,23]. In Gems, TDP-43 was found to interact with U snRNP proteins such as PRPF3 and U1-70K, and its depletion affected Gem integrity [22,91] (Figure 2B). Furthermore, Gem numbers were found reduced in a TDP-43 knockout mouse model [92], and TDP-43 loss led to abnormal U snRNA accumulation [22]. In a more recent study, TDP-43 has been found to regulate a class of regulatory RNAs, small CB specific RNAs (scaRNAs) involved in snRNA processing [93]. TDP-43 facilitates localisation of specific subpopulations of scaRNAs to CBs in WDR79 protein-dependent and -independent manner [93]. By promoting the localisation of C/D-type scaRNAs to CBs, TDP-43 indirectly regulates the site-specific 2′-*O*-methylation of U1 and U2 snRNAs and prevents the nucleolar redistribution of certain scaRNAs [93] (Figure 2B). Thus, the role of TDP-43 in RNA processing is complex, extending into the regulation of spliceosome assembly and maintenance, and some of these roles rely on its ability to be recruited into the nuclear condensates CBs/Gems.

### PML bodies

PML bodies are 0.1–10 µm subnuclear structures present in most cell types and tissues, although their numbers vary significantly [94]. The general feature of the proteins recruited to PML bodies is their ability to be sumoylated. These structures may be broadly involved in PTM regulation and protein quality control [95], and their assembly is enhanced by cellular stress [96]. A recent study has implicated the recruitment of TDP-43 and other RBPs into PML bodies in their protection from degradation during stress [24] (Figure 2C). A combination of SNAP-tag labelling and phase-specific fluorophores capable of detecting compartment-specific protein folding states have revealed that under proteotoxic stress, TDP-43 is sequestered into PML bodies in its native conformation, together with heat shock proteins such as HSPA1A. With prolonged stress however, TDP-43 undergoes conformational changes consistent with misfolding and potentially, loss of solubility [24] (Figure 2C). Being constitutive, PML bodies may be involved in maintaining TDP-43 homeostasis outside acute stress conditions, which must be addressed in future studies.

## TDP-43 in stress-induced nuclear condensates

During stress, the cell nucleus undergoes remodelling at all levels, including RBP redistribution, marked by reversible assembly of *de novo* condensates.

### Nuclear stress bodies

nSBs are granules of variable size (0.3–3 µm) assembled due to activated transcription of highly repetitive satellite III transcripts originating from heterochromatin, in response to a range of stressors such as thermal stress [97]. HSF1 is recruited onto these transcripts, alongside multiple other proteins, leading to its polymerisation and nuclear body assembly [98]. Proteomic analysis has revealed enrichment of nSBs for transcription and splicing factors, e.g. from hnRNP and SRSF families, including TDP-43 [99]. nSBs provide a platform for rapid changes in SRSF protein phosphorylation and intron retention controlled by these proteins [99]. TDP-43 condensates were previously detected in the nucleus of cells recovering from heat shock — identified as nSBs by co-localisation with SAFB protein, an established nSB marker [25]. LCD-containing proteins are tightly regulated by heat shock proteins, and a proteomics study confirmed TDP-43 interactions with several family members, e.g. HSP70 isoforms [100], further validated in follow-up functional studies [101]. The steady-state complex of TDP-43 with HSP70 and HSP40 was found to be dismantled during the recovery from heat stress, correlating with TDP-43 recruitment into nSBs, with HSP40/70 overexpression blocking this event [25]. In the process, TDP-43 also dissociated from another RBP — splicing factor hnRNPA1 [25]. RIP-seq analysis showed reduced TDP-43 binding to RNA, despite increased abundance of some transcript classes, such as those related to unfolded protein response (UPR) [25]. Collectively, these data point to a mechanism of functional TDP-43 inactivation by sequestration into nSBs under acute stress, leading to its transient loss of function in RNA processing (Figure 3A).

### Stress-induced nuclear TDP-43 condensates (TCs, TDP-43 NBs)

Earlier reports detected visible granulation of TDP-43 in response to arsenite stress and began characterising the structure and regulation of these granules [26,60,61]. In the two recent studies from our group, it has been demonstrated that these structures (abbreviated as ‘TCs’) are distinct from SAFB-positive nSBs and are critically involved in the regulation of splicing as well as another nuclear condensate, the paraspeckle [27,52] (Figure 3B). Mechanistically, HSP70 oxidation and loss of chaperoning function on TDP-43 may contribute to their assembly [53]. Such condensates can also form *in vivo*, in the CNS of mice, upon delivery of toxic nanoparticles [53]. Proteomic profiling has revealed that TC sequestration is associated with TDP-43 loss of interactions with multiple protein binding partners from the ‘gene expression’ and ‘RNA processing’ pathways. Along with the low complexity of the TC proteome, this suggested that TDP-43 transient inactivation might be their primary function. Furthermore, their assembly is accompanied by TDP-43 dissociation from RNA, such that TCs are depleted of (polyadenylated) RNAs, and correlates with splicing alterations consistent with TDP-43 loss of function [52]. Consistently, in another recent study, changes to TDP-43 regulated splicing were detected in cells exposed to an approved medication ciclopirox causative of heavy-metal toxicity and oxidative stress [102]. Importantly, stress-induced splicing changes are fully reversible, where TC dissolution coincides with restoration of splicing [52]. TDP-43 is a negative regulator of paraspeckles, as described above, and our detailed studies have revealed that TDP-43 becomes depleted from paraspeckles through its TC sequestration, which may contribute to their augmented assembly under stress [27] (Figure 3B). Notably, unlike nSBs that are nucleated and possibly, maintained by satellite III lncRNAs, TCs seem to be RNA-independent and are inhibited by (UG-rich) RNA [52].

To conclude, TCs and nSBs transiently sequester TDP-43 during stress, with measurable effects on RNA processing. Thus, cells employ more than one mechanism to achieve functional TDP-43 inactivation during stress, highlighting the physiological significance of this effect. Shutdown of the activity of TDP-43 — a key factor in RNA metabolism in the steady-state — may be essential for rerouting cellular resources to stress response programmes. In addition, these structures may serve as ‘storage’ condensates, synergising with PML bodies [24] in preventing TDP-43 degradation during stress.

Formation of TCs (and potentially, nSBs) is a conserved response seen in different cell types, from cancer cell lines to neurons, and for a variety of stressors [52]. However, TDP-43 can also form d*e novo* nuclear bodies in a context-specific manner (Table 1). For example, TDP-43 positive nuclear granules assemble in lipopolysaccharides (LPS)-stimulated macrophages and dendritic cells [103]. These structures, termed interleukin-splicing activating compartment (InSAC), contain interleukin pre-mRNA and snRNPs released from CBs. Interestingly, in these InSAC bodies, TDP-43 is ubiquitinated — a PTM usually associated with its non-functional states. Notably, formation of these foci was accompanied by CB disruption, in line with TDP-43's role in CB/Gem integrity [22]. Finally, transcriptional stress is associated with redistribution of RBPs including TDP-43 into structures on the surface of the nucleolus — ‘nucleolar caps’ [79,104]. These assemblies possess both liquid- and solid-like subdomains, contain pre-rRNA and may function as storage depots for proteins until the transcription is resumed [104].

Table 1 summarises the properties of the constitutive and stress-induced TDP-43 containing condensates, including their biophysical states and RNA content. TDP-43 containing constitutive condensates rich in RNA tend to possess more liquid-like properties, for example splicing condensates and paraspeckles are both sensitive to 1,6-hexanediol and highly dynamic by FRAP analysis [16,59]. On the other hand, stress-induced functional condensates of TDP-43 display higher stability, implicating more solid-like properties. For example, TCs contain β-sheet/amyloid structure, are resistant to 1,6-hexanediol and display slow recovery in FRAP experiments [52,53]. Alterations to the core properties of constitutive condensates, abnormal assembly of stress-induced MLOs and formation of *de novo* condensates are associated with TDP-43 pathological changes in neurodegeneration, as discussed below.

## Dysfunction of TDP-43 rich nuclear condensates in neurodegeneration

TDP-43 loss from the nucleus and its mislocalisation to the cytoplasm in the CNS cell types, sometimes accompanied by its aggregation (‘TDP-43 proteinopathy’), are observed in nearly all patients with sporadic amyotrophic lateral sclerosis (ALS), a large proportion of familial ALS and frontotemporal dementia (FTD), a subset of Alzheimer's disease, multisystem proteinopathy and myopathy cases [105,106]. In addition to the CNS, abnormal TDP-43 positive structures in the cytoplasm have been detected in the muscle and neuromuscular junctions in ALS and multisystem proteinopathy [17,107] and in microglia in traumatic brain injury [108]. The cytoplasmic TDP-43 pathology has been in a focus of neurodegeneration research since its discovery. However, the growing appreciation of the nuclear changes as early disease hallmarks should elicit a major shift in the TDP-43 proteinopathy studies in the upcoming years. Such refocusing is supported by the TDP-43 loss-of-function splicing signatures in postmortem tissue of ALS/FTD and Alzheimer's disease patients [109,110] and identification of TDP-43 nuclear granulation in ALS neurons using aptamer detection [111]. Gain of function in the nucleus by aberrant TDP-43 species, including changes to MLOs, in the absence of its cytoplasmic mislocalisation, represents another piece in the TDP-43 proteinopathy ‘puzzle’ [112].

### Altered TDP-43 condensation in disease

**Figure 4.**
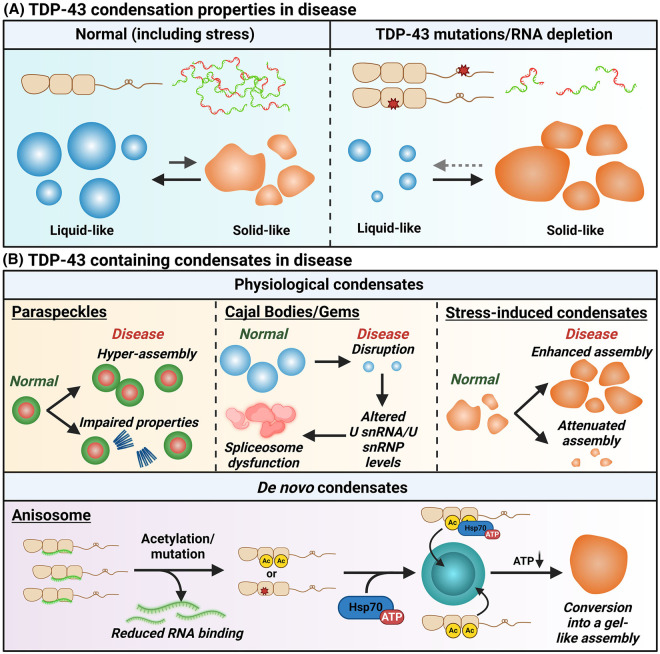
Dysregulation of TDP-43 containing nuclear condensates in neurodegeneration. (**A**) Changes to the properties of TDP-43 containing nuclear condensates in neurodegenerative disease. (**B**) Dysfunction of physiological TDP-43 containing condensates and possible *de novo* condensate assembly in ALS.

Although TDP-43 mutations are a rare cause of ALS (<1% of cases) [113], their studies have been highly instrumental in dissecting the role of its condensation in neurodegenerative disease. The CTD of TDP-43 is a hotspot of ALS mutations, and many of them map to CR [14]. CR-breaking variants disrupt TDP-43 LLPS, decreasing the fluidity of assemblies *in vitro* and in cells, and impair condensation-dependent splicing [16,42,47]. Reflecting the antagonism between LLPS and solid-like condensation, these mutations enhance TDP-43 incorporation into more solid-like assemblies such as TCs [47,52]. Intriguingly, rare TDP-43 NTD-affecting mutations that promote LLPS and reduce aggregation [26,114] have an opposite effect on TC partitioning [52]. Thus, disturbing TDP-43's ability to switch between phase separation states is detrimental regardless of the direction of change. Decreased RNA availability driven by altered transcription rates [115] and enhanced RNA degradation [116], mutations disrupting RNA binding [117], and aberrant PTM profiles [60] will all impact the RNA interactome of TDP-43, thereby shifting the balance towards RNA-depleted, less fluid assemblies (Figure 4A). Higher prevalence and prolonged maintenance of such assemblies will eventually exhaust cellular disassembly mechanisms, increasing the likelihood of irreversible aggregation in the nucleus [118,119]. Indeed, nuclear TDP-43 aggregates have been detected in disease [120]. Furthermore, dysfunction of a subset of condensates can destabilise the entire condensate network [121]. For example, attenuated TC formation will affect cytoprotective paraspeckle hyper-assembly under stress [27,82].

### Physiological nuclear condensates in disease

Multiple lines of evidence point to wide-spread alterations of TDP-43 containing nuclear condensates in ALS, including studies in disease models and post-mortem tissue (Figure 4B). Paraspeckle hyper-assembly is typical for ALS spinal cord motor neurons, irrespective of the disease subtype [122–124]. Structural variations in NEAT1_2 may enhance paraspeckle suppression by TDP-43, leading to attenuated stress response and neuronal vulnerability and ultimately, resulting in more severe disease [27]. Being LLPS-based [59], paraspeckles are likely structurally and functionally impaired by TDP-43 mutations that modulate its phase-separating properties, although it is yet to be proven experimentally. Diminished CB/Gem numbers were reported in neurons of a transgenic TDP-43 mouse model [92] — a phenotype subsequently confirmed in ALS cellular models and postmortem tissue [22,91]. Mechanistically, TDP-43 dysfunction leads to aberrant accumulation of U snRNPs in ALS motor neurons, which is presumably responsible for spliceosome collapse [22]. TDP-43 coexists with FUS in many RNP complexes, including U snRNPs [125], and disturbed stoichiometry of the two proteins in the nucleus due to altered condensation will contribute to neuronal dysfunction. A recent study has reported loss of PML bodies in ALS spinal cord and brain neurons [126], which, given their protective role against TDP-43 degradation during stress [24], can contribute to its depletion. Finally, stress-inducible TDP-43 condensates are highly sensitive to TDP-43 mutations, where different variants can either augment or attenuate TC assembly [26,52]. Enhanced TC formation associated with prolonged TDP-43 retention and inactivation would extend splicing loss-of-function and paraspeckle hyper-assembly. In sporadic ALS cases, where external factors are contributory, chronic stress exposure may cause recurring TC assembly and impact slowly recovering splicing targets such as STMN2, thereby phenocopying the effect of TDP-43 mutations [52].

### De novo TDP-43 condensation in disease

TDP-43 deficient in RNA binding, due to a mutation affecting RRM or a specific PTM(s), is prone to excessive condensation in the nucleus [70,117,127]. Intriguingly, we found that nuclear TDP-43 granulation can be triggered by the condensation of poly-PR peptide derived from ALS/FTD-linked *C9orf72* gene [128]. Mutant TDP-43 nuclear condensates can have an ‘amorphous’ appearance [117] or alternatively, ordered arrangement [69,129]. For example, disease-linked acetylated TDP-43 can phase separate into anisotropic droplets with a dense but dynamic shell and liquid centre — ‘anisosomes’ [69] (Figure 4B). The structures are dynamic, ATP-dependent and enriched in HSP70 proteins in their core, however they can lose fluidity with time, solidify and convert into an aggregate [69]. Intriguingly, our recent study has identified RNA-containing anisotropic condensates in the nucleus as intermediates of C9orf72 poly-PR aggregation [128]. Another protein associated with neurological diseases, DDX3X, was reported to form cytoplasmic hollow-centre condensates, potentiated by impaired RNA binding [130]. The unstable, dynamic nature of anisotropic condensates may preclude their detection in postmortem tissue and even in *in vivo* models, however they may mark the earliest disease stages and represent a common phenotype in neurodegeneration. Further studies are warranted to establish whether such LLPS condensates of TDP-43 and other proteins are indeed relevant to human disease and if so, whether they are protective or pathological.

## Outlook

TDP-43 is an important house-keeping protein essential for normal cell function. Selective susceptibility of certain cell types to its dysmetabolism remains enigmatic. Neurons typically affected in TDP-43 proteinopathies may be characterised by specific TDP-43 assembly states that increase their vulnerability to cellular stress and other disease risk factors. For example, we have detected high basal levels of non-oligomerised TDP-43 specifically in neurons, which may delay the protective TDP-43 condensation during stress [52]. Profiling the cell type-specificity of TDP-43 self-assembly patterns and condensate biophysical properties may therefore provide clues on the differential susceptibility to TDP-43 proteinopathy. Given the non-LLPS, amyloid-like nature of some physiological TDP-43 condensates, a crucial question remains as to how these condensates are resolved and whether their clearance processes are impaired in disease. Finally, TDP-43 nuclear and cytoplasmic condensation are intimately linked. For example, TDP-43 binding to RNA and its ability to assemble higher-order RNP complexes have been found to prevent its cytoplasmic efflux in the steady-state and under stress [52,131,132]. Alterations of TDP-43 containing nuclear condensates constitute the earliest disease pathology and with further research, should become a source of targets for timely therapeutic intervention.

## Perspectives

- TDP-43, a key player in neurodegeneration, is a nuclear RNA-binding protein and splicing factor. It is a known component of phase-separated assemblies, however we are only beginning to decrypt its roes in the nuclear condensates.
- Depending on the mode of its homotypic interactions and the repertoire of RNAs it binds, TDP-43 modulates the properties and function of nuclear condensates, both constitutive and stress-induced. Nuclear condensates, in their turn, can regulate TDP-43 availability.
- Structural alterations in TDP-43, its PTMs or stress-induced changes that compromise its association with nuclear condensates may underlie the earliest cellular pathologies in neurodegenerative disease.

## Open Access

Open access for this article was enabled by the participation of University of Sheffield in an all-inclusive *Read & Publish* agreement with Portland Press and the Biochemical Society under a transformative agreement with JISC.

## Abbreviations

1,6-HD: 1,6-hexanediol
ALS: amyotrophic lateral sclerosis
CB: Cajal body
CNS: central nervous system
CR: conserved region
CTD: C-terminal domain
FRAP: fluorescence recovery after photobleaching
FTD: frontotemporal dementia
LARKS: low-complexity aromatic-rich kinked segments
LCD: low-complexity domain
LLPS: liquid-liquid phase separation
lncRNA: long non-coding RNA
MLO: membraneless organelle
nSB: nuclear stress body
NTD: N-terminal domain
PML: promyelocytic leukaemia
Poly-PR: proline-arginine dipeptide repeat
PTM: post-translational protein modification
RBP: RNA-binding protein
RNP: ribonucleoprotein
scaRNA: small Cajal body specific RNA
snRNP: small nuclear ribonucleoprotein
TC: stress-induced nuclear TDP-43 condensate
UPR: unfolded protein response
